# Bioactive Natural Product and Superacid Chemistry for Lead Compound Identification: A Case Study of Selective hCA III and L-Type Ca^2+^ Current Inhibitors for Hypotensive Agent Discovery

**DOI:** 10.3390/molecules22060915

**Published:** 2017-05-31

**Authors:** Hélène Carreyre, Grégoire Carré, Maurice Ouedraogo, Clarisse Vandebrouck, Jocelyn Bescond, Claudiu T. Supuran, Sébastien Thibaudeau

**Affiliations:** 1 Superacid Group/Organic Synthesis Team, Université de Poitiers, IC2MP—UMR CNRS 7285, 86073 Poitiers CEDEX 09, France; helene.carreyre@univ-poitiers.fr; 2 STIM—ERL CNRS 7368 Université de Poitiers, 86073 Poitiers Cedex 9, France; gregoire.carre@etu.univ-poitiers.fr (G.C.); clarisse.vandebrouck@univ-poitiers.fr (C.V.); jocelyn.bescond@univ-poitiers.fr (J.B.); 3 Laboratoire de Physiologie Animale, Université de Ouagadougou, 03 BP 7021 Ouagadougou 01, Burkina Faso; ouedraogo_maurice@yahoo.fr; 4 Department of Neurofarba, Sez, Chimica Farmaceutica e Nutraceutica, University of Florence, 50019 Sesto Fiorentino, Italy

**Keywords:** dodoneine, carbonic anhydrase, L-type calcium channel, molecular diversity, superacid

## Abstract

Dodoneine (Ddn) is one of the active compounds identified from *Agelanthus dodoneifolius*, which is a medicinal plant used in African pharmacopeia and traditional medicine for the treatment of hypertension. In the context of a scientific program aiming at discovering new hypotensive agents through the original combination of natural product discovery and superacid chemistry diversification, and after evidencing dodoneine’s vasorelaxant effect on rat aorta, superacid modifications allowed us to generate original analogues which showed selective human carbonic anhydrase III (hCA III) and L-type Ca^2+^ current inhibition. These derivatives can now be considered as new lead compounds for vasorelaxant therapeutics targeting these two proteins.

## 1. Introduction

The narrowing of therapeutic focus, the expansion of biotechnologies and bio-based therapies, and the necessity to hit the target not only accurately but very quickly are trends that characterize the present pharmaceutical environment [1,2]. In this context, and despite the fantastic progress made after the human genome characterization in the field of biotechnology [3], small molecules remain central players in medicinal chemistry [4,5,6]. Thus, the generation of active drug molecules is currently a highly important part of drug discovery, and novel synthetic and screening methodologies continue to be explored to face this worthy challenge. The first expectation, yet unfulfilled, that combinatorial chemistry techniques would provide all the chemicals needed for lead discovery has progressively led to the exploitation of new strategies, such as target-guided synthesis, fragment-based drug discovery, and diversity-oriented synthesis [7,8,9,10,11]. There is also a growing powerful case for re-exploring natural products for drug discovery [12,13,14]. Between 1981 and 2010, 34% of new medicines approved by the US Food and Drug Administration (FDA) were natural products or direct derivatives of natural products [15]. The wide range of pharmacophores and the high degree of stereochemistry furnished by natural products collection may account for the re-emergence of natural products for drug discovery, offering biologically relevant “chemical space” [16,17]. In addition, natural products can serve to reveal novel aspects of physiology with strong incidences for therapeutics discovery in underexplored biological space [18]. To fully exploit the potential of natural products, chemo and site-selective chemical modifications are required to tailor physicochemical properties, to modify metabolism, or to improve the ADME (Absorption, Distribution, Metabolism, and Excretion) properties or selectivity of a drug [19]. To this end, late-stage functionalization is recognized as an especially efficient strategy and the available synthetic arsenal for molecular function optimization is growing tremendously [20,21]. In superacid conditions [22], polyfunctionalized molecules can be polyprotonated and react through an original mode of activation (superelectrophilic activation) [23] that allows for direct modification in positions that cannot be accessible by using conventional media. Under these conditions, natural products can be selectively and directly functionalized (ketones, phenols, terpenes, steroids, alkaloids) [24,25] to generate new bioactive compounds. For example, this strategy was shown to be especially efficient for the discovery of a new anticancer agent (JAVLOR^®^) derived from *Vinca* alkaloids [26].

Here, we report recent data obtained in the context of a scientific program aiming at discovering new hypotensive agents through the original combination of natural product discovery and superacid chemistry diversification. A natural product, dodoneine, was isolated from an African hemi-plant parasite used as a remedy to treat cardiovascular and respiratory diseases. After evidencing its vasorelaxant effect on rat aorta, superacid modifications allowed us to generate original analogues which showed selective human carbonic anhydrase III (hCA III) and L-type Ca^2+^ current inhibition and can now be considered as new lead compounds for vasorelaxant therapeutics synergistically targeting two proteins.

## 2. Results

### 2.1. Structure Elucidation of a Dihydropyranone from Tapinanthus dodoneifolius

**Figure 1:**
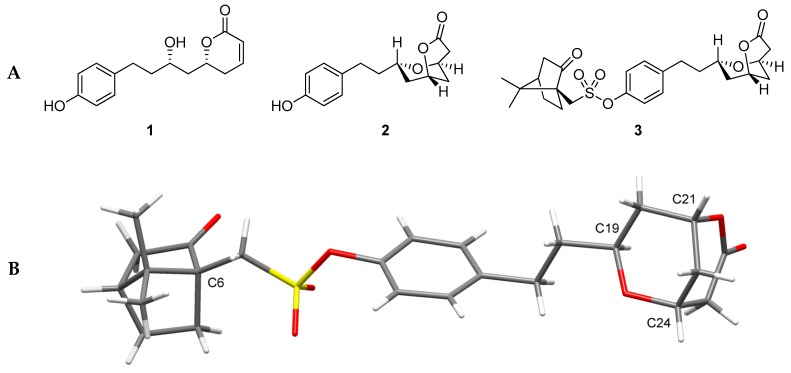
(**A**) Dodoneine **1** and its modification to a bicyclic lactone **2** and its camphorsulfonate analogue **3**; (**B**) X-ray structure of camphor sulfonate **3**.

Hypertension is one of the most common diseases in the world. Epidemiologic data indicated that 26.4% of the adult population had hypertension in 2005 and that 29.2% were projected to have this condition by 2025 [27]. In developing countries, where it also affects a significant part of the population [28], hypertension is usually treated by plant decoctions or extracts such as infusions of *Agelanthus dodoneifolius*. Ouedraogo et al. [29] showed that the crude aqueous extract (AE) and the ethanolic extract (EE) of *Agelanthus dodoneifolius* inhibited acetylcholine-induced bronchoconstriction on rat trachea. It was also reported that the crude aqueous extract had a vasorelaxant effect on rat aorta. When this study started in 2006, the presence of tannins, anthracenosides, anthraquinones, alkaloids, saponins, sterols, and triterpenes were shown to be detected in the plant. Using an accelerated solvent extractor apparatus at 60 °C under pressure, a methanolic extract was obtained. Its tested physiological activity was shown to be analogous to the whole plant activity. Thin layer chromatography analysis (TLC) revealed the presence of one main compound, which after slow crystallization from petroleum ether/toluene afforded one compound existing as a sole dextrorotary enantiomer, as indicated by polarimetry and chiral liquid chromatography. After extensive nuclear magnetic resonance (NMR) and mass spectrometry experiments (ESIMS), combined with infrared spectroscopy analysis (FT-IR), the structure was revealed to be a new dihydropyranone, (*R*)-6-[(*S*)-2-hydroxy-4-(4-hydroxyphenyl)butyl]-5,6-dihydropyran-2-one, named dodoneine (**1**) (Figure 1A). In the biphasic system water/CH_2_Cl_2_ containing K_2_CO_3_, compound **1** could be converted to a bicyclic lactone **2,** which afforded **3** after treatment with (1*S*)-(+)-10-camphorsulfonyl chloride. X-ray crystallographic analysis of **3** (Figure 1B), thanks to the known configuration of the camphor sulfonate moieties, allowed for the absolute configuration identification of every asymmetric carbon and confirmed the structure of **1** (Figure 1A) [30].

### 2.2. Hypotensive Effect of Dodoneine

**Figure 2:**
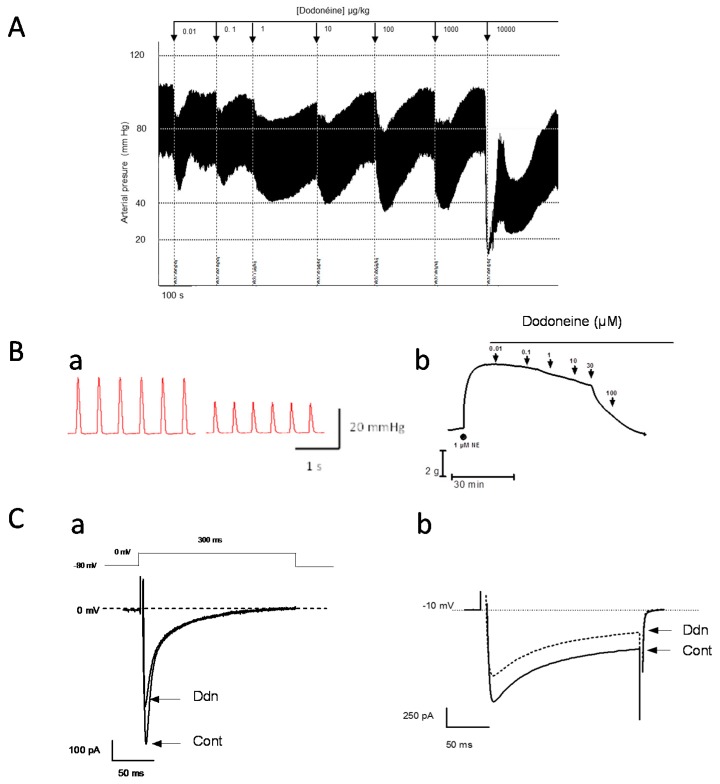
Physiological effects of dodoneine in vivo, ex vivo, and in vitro: on anesthetized rat carotidal blood pressure (**A**); on the left ventricular developed pressure (LVDP) of isolated perfused rat heart (**Ba**); on rat precontracted aortic rings (**Bb**); on the L-type calcium current recorded in cardiomyocytes (**Ca**); and on A7r5 smooth muscle cells line (**Cb**). Barium was used in substitution of calcium to record the current.

To estimate the hypotensive effect of dodoneine, due to quantify limitations, a first screen of ethanolic extract fractions revealed that one of the CH_2_Cl_2_/AcOEt fractions was the fraction showing the highest concentration of the bioactive compound dodoneine, and was thus used for ex vivo and in vivo vasorelaxant activity evaluation and then compared to the whole plant activity and to pure dodoneine activity. In anesthetized normotensive rats, successive intravenous injections of CH_2_Cl_2_/AcOEt fraction (0.01–10 mg/kg) produced a dose-dependent decrease in both systolic and diastolic pressure. This hypotensive effect on rat arterial pressure was confirmed ex vivo; the cumulative application of CH_2_Cl_2_/AcOEt fraction (0.001–3 mg/kg) into the organ induced a concentration-dependent relaxation on rat precontracted aortic rings. Dodoneine separated from this fraction also induced a concentration-dependent aortic relaxation effect with an IC_50_ value of 81.4 µM [30], and dodoneine was able to produce a hypotensive effect on the whole body (Figure 2A,B); the decrease of carotidal pressure values recorded in normotensive rats was about 35% at the highest dose tested (100 µg/kg) without any significant effect on heart rate [31]. This hypotensive effect of dodoneine following an acute administration on anesthetized rats has also been observed when the blood pressure has been measured after chronic treatment (decrease of the systolic values by 20% for dodoneine at 20 µg/kg per day during 15 days), confirming the use of the plant decoction in traditional African medicine to treat hypertension.

### 2.3. Hypotensive Properties of Dodoneine are Likely Associated with a Negative Inotropic Effect and L-Type Calcium Current Inhibitor

This hypotensive effect could be associated not only with the vasorelaxant effect of dodoneine producing the decrease of peripheral resistance to blood circulation in the whole body, but also to a direct cardiac effect. Ex vivo on isolated perfused rat heart, it has been demonstrated that dodoneine significantly decreased the left ventricular developed pressure (LVDP) in a dose-dependent manner with the half maximal response (IC_50_) value of 9.8 µM (Figure 2Ba) [32]. Then, in addition to known hypotensive molecules diminishing blood pressure [33], dodoneine exhibited vasorelaxant and negative inotropic effects. Regarding these effects, the hypothesis of the regulation of the calcium signaling by dodoneine on vascular and cardiac muscular cells emerged [34]. Mechanistic studies have been performed at cellular levels in order to determine a molecular target. First, the effects of dodoneine were characterized in freshly dissociated rat cardiac ventricular myocytes using the whole cell patch-clamp configuration of the electrophysiological techniques usually used to record the activity of membrane ionic channels involved in the physiology of excitable cells. In these experimental conditions, dodoneine dose-dependently inhibited the L-type calcium current, an ionic current mainly involved in the excitation contraction coupling in muscular cells, with an IC_50_ of 3.2 µM. The others characteristics (kinetic of activation and inactivation; frequency-dependent effects) of the blockade of the calcium current have indicated that dodoneine has its own specific properties when compared to the classical calcium channel blockers generally used in the treatment of hypertension. In a second experiment, the inhibition of the L-type calcium current by dodoneine was confirmed on smooth muscle cells (by about 30% at 100 µM, as on ventricular cardiomyocytes), but with less efficiency than the calcium channel blocker (CCB) verapamil [35] (Figure 2C). Regarding these properties in the modulation of calcium cycling via the inhibition of calcium influx via the L-type calcium channel in vascular muscle cells and cardiac ventricular cells, dodoneine was identified to be a new natural calcium blocker able to decrease blood pressure with combined vasorelaxant and negative inotropic effects as other CCBs [36].

### 2.4. Dodoneine and Its Analogues Also Inhibit Human Carbonic Anhydrases

Even if important remaining gaps relating to the treatment of hypertensive-related diseases with human carbonic anhydrases inhibitors exist, considerable evidence points to vasodilation by carbonic anhydrase inhibitors (CAI) [37]. The vasodilator effects of thiazide diuretics were shown to result primarily from the inhibition of vascular smooth muscle cell carbonic anhydrase [38], and carbonic anhydrase (CA) was also shown to be implicated in mediating the hypertrophy response of cardiac myocyte to phenylephedrine [39], which suggests that CA inhibition could represent an effective therapeutic approach against heart failure. Carbonic anhydrases (EC 4.2.1.1) are widespread enzymes in all organisms. These zinc metalloenzymes catalyze CO_2_ hydration to bicarbonate and protons [40,41,42]. CO_2_, bicarbonate, and protons are essential molecules/ions in many important physiological/pathologic processes. Many mammalian CAs are well established therapeutic targets, with the potential to be inhibited to treat a wide range of disorders, including glaucoma, epilepsy, obesity, hyperglycemia, and more recently cancer [43]. In the design of new drugs, the selective inhibition of CAs has become an essential parameter, especially considering the high number of isozymes in mammals and their widespread distribution in the organism [43]. Designing inhibitors that interact with other parts of the enzyme besides the classical zinc-binding zone is now considered an especially relevant strategy to attain good selectivity [44]. This strategy led to the discovery of new chemo types that selectively inhibit some targeted hCA isoforms. For instance, coumarins, thiocoumarines [45,46,47], and more recently sulfocoumarins [48], located at the entrance of the enzyme active site, were found to act as selective inhibitors of several isoforms, such as hCA IX isozyme, a validated anti-cancer target. Phenol derivatives [49] and lactones [50] are also classically targeted hCA inhibitors, found to be low micromolar CAIs. Phenol was found anchored to the Zn (II)-coordinated water molecule, binding through two hydrogen bonds to this molecule and the OH of Thr199 [51], and can be considered as a non-zinc binding inhibitor.

**Figure 3:**
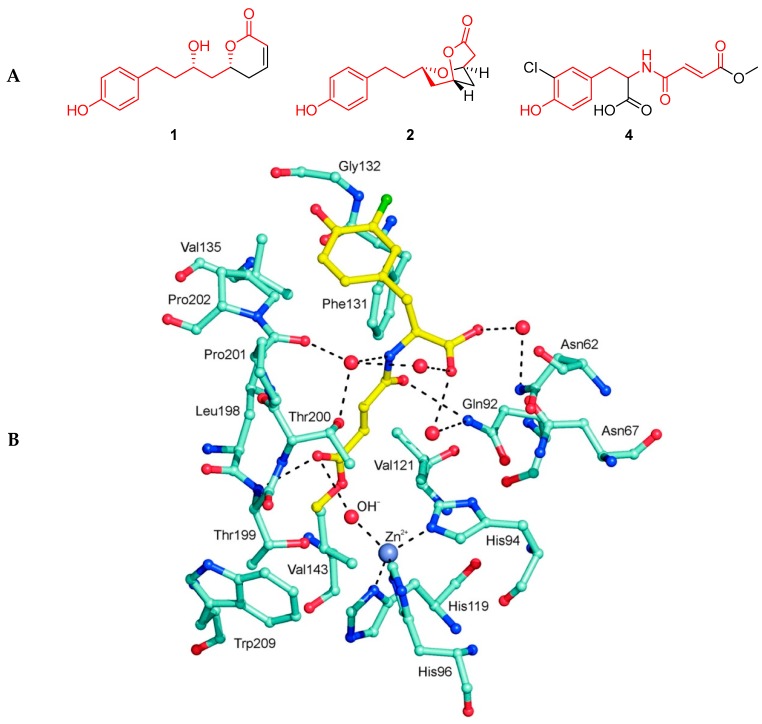
(**A**) Dodoneine **1**, its analogue **2**, and xylariamide A **4**; (**B**) Active site view of hCA II/**4** adduct (Protein Data Bank (PDB) code 3P4V) showing residues participating in the recognition of the inhibitor molecule. Only one conformation of the chlorophenol moiety is reported. Water molecules are shown as red circles.

In this context, it was especially interesting to note that dodoneine incorporates both phenol and lactone chemo types (Figure 3A). In addition, among the natural phenolic compounds known to act as carbonic anhydrase, compound **4** (xylariamide A) is a phenolic compound which acts as a micromolar hCA II inhibitor (K_i_ = 8 µM), which was especially intriguing. After co-crystallization with hCA II, the main hCA II/**4** interactions were identified after the analysis of its structures (Figure 3B) [52].

Compound **4** is anchored to the zinc-bound water molecule, not through the OH phenolic moiety but by means of a hydrogen bond involving the carbonyl oxygen of the ester functionality, which also makes a second hydrogen bond with the Thr199NH atom. The methyl moiety of the ester functionality is in van der Waals contact with Trp209. Various oxygen/nitrogen atoms of the inhibitor make additional direct or solvent-mediated hydrogen bonds with several enzyme residues, such as Thr200, Gln92, Pro201, and Asn62, whereas the chlorophenol moiety was slightly disordered (two conformations were observed), making only van der Waals contacts with the CA II active site. As represented in Figure 3A, dodoneine **1**, its analogue **2,** and xylariamide A **4** show structural and functional similarities. In addition, in xylariamide A, the locked-size chain (π-electron conjugation through the amido-substituted Michael ester) must be preponderant for hCA II inhibition, as shown by the abovementioned X-ray analysis. In dodoneine **1** and in compound **2**, the dihydropyranone core and the bicyclic lactone cores, respectively, must be also locked. Then the question arises: could the hypotensive effect of dodoneine also be related to the selective inhibition of human carbonic anhydrases vascular smooth muscle cells?

Initially, dodoneine **1** was tested against all the catalytically active mammalian CA isoforms, hCA I–XIV (Table 1). Dodoneine showed inhibition in the range of 5.5–10.4 µM against isoforms I, III, IV, XIII, and XIV, and did not inhibit the other isoforms. To the best of our knowledge, in terms of selectivity, the inhibitory profile of this compound is original.

**Scheme 1:**
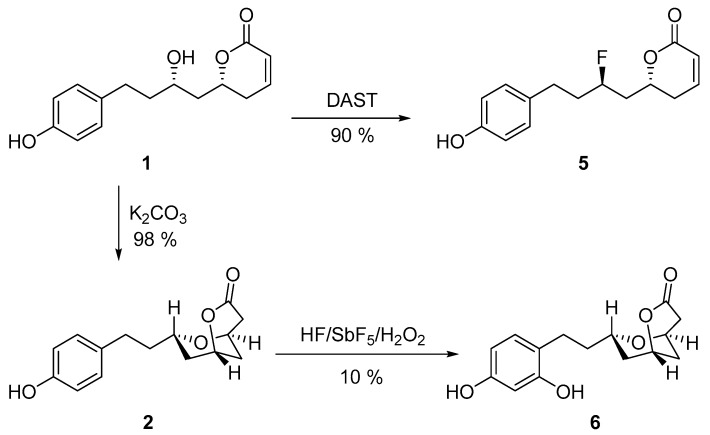
Chemical modifications of dodoneine **1** leading to derivatives **2**, **5**, and **6**.

Especially interesting was the selective inhibition of human isoform III toward the widespread and highly catalytically active isoform II. As mentioned above, hCA I, hCA II, and hCA III are present in mammalian vascular smooth muscle, and their activities contribute to vasoregulation [54]. Interestingly, dodoneine analogue **2** presented a similar inhibition profile to dodoneine, without inhibiting hCA I, and thus showed a very strong selectivity for hCA III and mCA XIII (Scheme 1).

Compound **2**, easily obtained from dodoneine in aqueous solution, could be considered as being in vivo generated from dodoneine. Back to the dodoneine extract, we identified this bicyclic analogue of dodoneine to be present in low quantities in the methanolic extract. This confirmed our hypothesis and encouraged us to verify whether the chemotype responsible for hCA III selective inhibition would not be the whole structure **2** instead of doneine itself. To this end, the alcohol position was locked by generating its fluoro-analogue **5**. Due to the C–F bond properties, [55,56] blocking metabolic position with fluorine atom is a common practice in medicinal chemistry research [57], and our group has already contributed to this field [58,59,60,61,62]. Compound **5** was then tested as an hCA inhibitor and its inhibition profile was completely different from dodoneine **1** and compound **2**, thus confirming that phenolic bicycle **2** can be considered as a new lead structure in the quest for hCA III selective inhibitors [63]. As a proof of concept, exploiting superacid chemistry, hydroxylated analogue **6** was generated directly from the chiral lead compound **2** [63]. Its inhibition profile, similar to compound **2**, encourages further studies in this direction.

**Figure 4:**
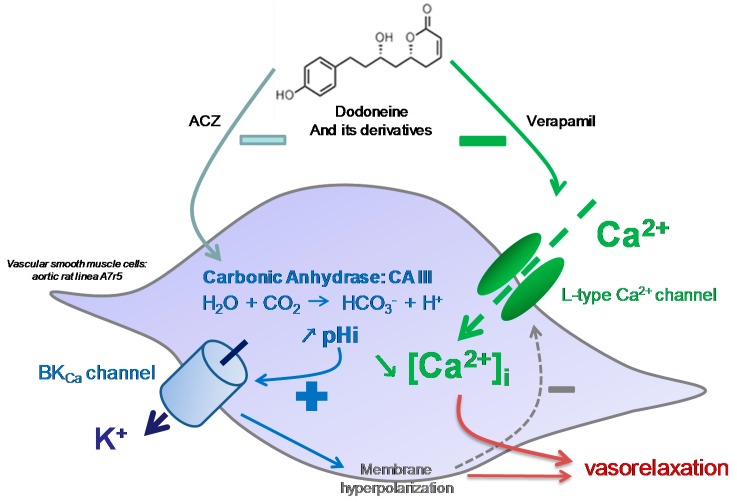
A proposed model for the dual inhibition of the L-type calcium channel and carbonic anhydrase by dodoneine in smooth muscle cells, leading to the vasorelaxation involved in reduced blood pressure (+: activation; −: inhibition). ACZ: acetazolamide, pHi: intracellular pH; BK_Ca_: large conductance calcium activated potassium channels.

To further evaluate the hypothesis that dodoneine **1** and its metabolite **2** induce vasorelaxation through a synergistic inhibition of the calcium channel current and selective carbonic anhydrase III, the effect of dodoneine was investigated on vascular smooth muscle at tissue and cellular levels. First, the molecular identities of CA isozymes were examined with reverse transcription polymerase chain reaction (RT-PCR): isozymes II, III, XIII, and XIV were present in rat aorta, while only the isozymes III, XIII, and XIV were expressed in the A7r5 smooth muscle cells line [35]. With specific pharmacological tools targeting CA and the L-type calcium channel (acetazolamide (ACZ) and verapamil, respectively), the efficiency of the blockade of the two pathways by dodoneine was verified on the vascular response. Previously, it has been shown that verapamil, as dodoneine, blocked the L-type calcium current, while ACZ did not modify this current. On the other hand, ACZ, as dodoneine, modified some mechanisms of a cellular transduction pathway involving CA (intracellular pH (pH_i_), large conductance calcium activated potassium channels (BKCas), and membrane potential). In fact, we demonstrated that the vasorelaxant effect of ACZ and dodoneine is a consequence of CA inhibition which leads to intracellular alkalinization, involved in BK_Ca_ channels activation and membrane hyperpolarization (Figure 4). Then, we checked the blockade of the two pathways independently and in combination. When the two controls, verapamil and ACZ, were applied together to inhibit calcium channels and CA, respectively, their relaxant effect on vasoconstricted aortic rings was cumulative. When dodoneine was applied concomitantly with one of these two controls, the vasorelaxant effect was higher than the effect obtained for dodoneine, verapamil, or ACZ alone, indicating that when one of the two targets is blocked, dodoneine is able to increase vasorelaxation by acting via the inhibition of the second target.

## 3. Conclusions

In conclusion, we demonstrated that the natural product dodoneine metabolite can be considered as a lead compound in the quest for therapeutics for hypertension. This study also demonstrated that, in aortic smooth muscle cells, these inhibitors act as L-type calcium channel blockers and hCA III inhibitors. Exploiting superacid chemistry was shown to be especially efficient to directly generate analogues through late-stage modification of these bioactive leads, and further studies are currently ongoing in this direction.

## Figures and Tables

**Table 1:** Human Carbonic Anhydrase inhibition data of dodoneine and its analogues (stopped flow CO_2_ hydrase assay) [53].

Compound	K_i_ (µm) ^a^											
	hCA I	hCA II	hCA III	hCA IV	hCA Va	hCA Vb	hCA VI	hCA VII	hCA IX	hCA XII	mCA^c^ XIII	hCA XIV
1	5.48	- ^b^	10.35	9.61	- ^b^	- ^b^	- ^b^	- ^b^	- ^b^	- ^b^	9.27	9.34
2	- ^b^	- ^b^	10.80	- ^b^	- ^b^	- ^b^	- ^b^	- ^b^	- ^b^	- ^b^	0.91	- ^b^
5	0.13	36.9	- ^b^	5.36	7.13	1.36	- ^b^	24.9	3.57	1.48	0.96	2.44
6	- ^b^	- ^b^	5.13	- ^b^	- ^b^	- ^b^	- ^b^	- ^b^	- ^b^	- ^b^	0.34	- ^b^

^a^ Errors in the range of ±5% of the reported data from three different assays; ^b^ not active >100; ^c^ mCA, murine Carbonic Anhydrase.
